# Clinical results of an arthroscopic modified Brostrom operation with and without an internal brace

**DOI:** 10.1007/s10195-016-0406-y

**Published:** 2016-04-23

**Authors:** Jae-Sung Yoo, Eun-Ah Yang

**Affiliations:** 1 Department of Orthopedic Surgery, Chungpyung Army Hospital, 926, GyungChoon-ro Chungpyung myun, Gapyung, GaPyung gun 477-815 South Korea; 2 Department of Anesthesiology, Cha Hospital, Bundang, South Korea

**Keywords:** Ankle, Instability, Reconstruction, Arthroscopy

## Abstract

**Background:**

The concept of utilizing nonabsorbable suture tape fixed directly to bone to augment Brostrom repairs of the anterior talofibular ligament (ATFL) has been proposed. However, no clinical data are currently available regarding the arthroscopic modified Brostrom operation with an internal brace.

**Materials and methods:**

This study involved 85 consecutive patients (22 in the with internal brace group; 63 in the without internal brace group) who could be followed up for >6 months after undergoing an arthroscopic modified Brostrom operation. The American Orthopaedic Foot & Ankle Society (AOFAS) score was administered to assess the functional status. At preoperation and at 24 weeks after surgery, the anterior drawer test was examined clinically.

**Results:**

Improvement of mean AOFAS score in the internal brace group from before surgery to two weeks after surgery was statistically significant (*p* < 0.05). At 24-week follow-up, the anterior drawer test showed grade 0 laxity in 19 patients (86.4 %) and grade 1 in three patients (13.6 %). Improvement of AOFAS score in the group without an internal brace from before surgery to 6 weeks after surgery was not statistically significant (*p* = 0.001). At 24-week follow-up, the anterior drawer test showed grade 0 laxity in 54 patients (85.7 %) and grade 1 in nine patients (14.3 %).

**Conclusion:**

Patients in the internal brace group were able to quickly return to activity and sports. We believe this technique could be a viable option for surgically treating chronic lateral ankle instability in patients who need an early return to activity and sports.

**Level of evidence:**

III.

## Introduction

Lateral ankle instability is a common pathological condition in recreational and professional athletes [1].

Most foot and ankle surgeons perform an open modified Brostrom operation for treatment of lateral ankle instability, and good-to-excellent results have been reported [2, 3].

Despite the value of the Brostrom procedure, limitations of this technique exist. Waldrop et al. [4] reported that both direct suture repair of the anterior talofibular ligament (ATFL) and the use of suture anchors in the fibula or talus had significantly inferior strength compared with the intact ATFL in a cadaveric model. As a result, the need for early protection of all three types of Brostrom procedures and cautious early rehabilitation were emphasized [4]. Kirk et al. [5] also recommended the need for protection to prevent ATFL elongation. Furthermore, in patients with long-standing lateral ankle instability with attenuated native tissue and in very large patients or athletes, both of whom are likely to place extra stress on their ankles, the adequacy of these repairs has been questioned [6, 7]. To address situations such as these, the concept of using high-strength nonabsorbable suture tape has been proposed, as described in previous literature for rotator cuff repairs [8, 9]. An internal brace is a ligament repair bridging concept using braided ultra-high-molecular-weight polyethylene/polyester suture tape and knotless bone anchors to reinforce ligament strength as a secondary stabilizer after repair and return to sports, which may help resist injury recurrence [10].

Almost exclusively, concomitant intra-articular ankle pathology is present and often best managed via an arthroscopic approach [11–13]. Recently a technique was developed to manage both the ancillary intra-articular pathology and the lateral ankle instability arthroscopically [14]. However, no clinical data are currently available regarding the arthroscopic modified Brostrom operation with an internal brace in the ankle.

The purpose of this study was to evaluate the clinical results of an arthroscopic modified Brostrom operation with an internal brace through comparison with an arthroscopic modified Brostrom operation without an internal brace. We hypothesized that an arthroscopic modified Brostrom operation with internal bracing could be useful for early rehabilitation and obtaining satisfactory clinical results.

## Materials and methods

This study was granted exemption by our Institutional Review Board. This study involved 85 consecutive patients (22 in the with internal brace group; 63 in the without internal brace group) who could be followed up for >6 months after undergoing an arthroscopic modified Brostrom operation at our hospital from April 2014 to July 2014. The average follow-up period was 7.4 months (6–9 months), the average age was 23 years (19–44), and all the patients were male soldiers because this institution is an army hospital.

Inclusion criteria were grade >2 mechanical laxity on the clinical and radiographic anterior drawer test and >2 episodes of functional instability (giving way) of the ankle. All patients were unresponsive to nonsurgical measures such as rest, bracing, anti-inflammatory drugs, proprioceptive training, ankle strengthening, and physical therapy for at least 6 months. Patients with systemic diseases, neuromuscular disorders, obesity and anatomic deformities, combined osteochondral lesion of the talus and previous surgery on the affected ankle were excluded.

### Surgical technique (arthroscopic modified Brostrom operation with an internal brace)

**Fig. 1:**
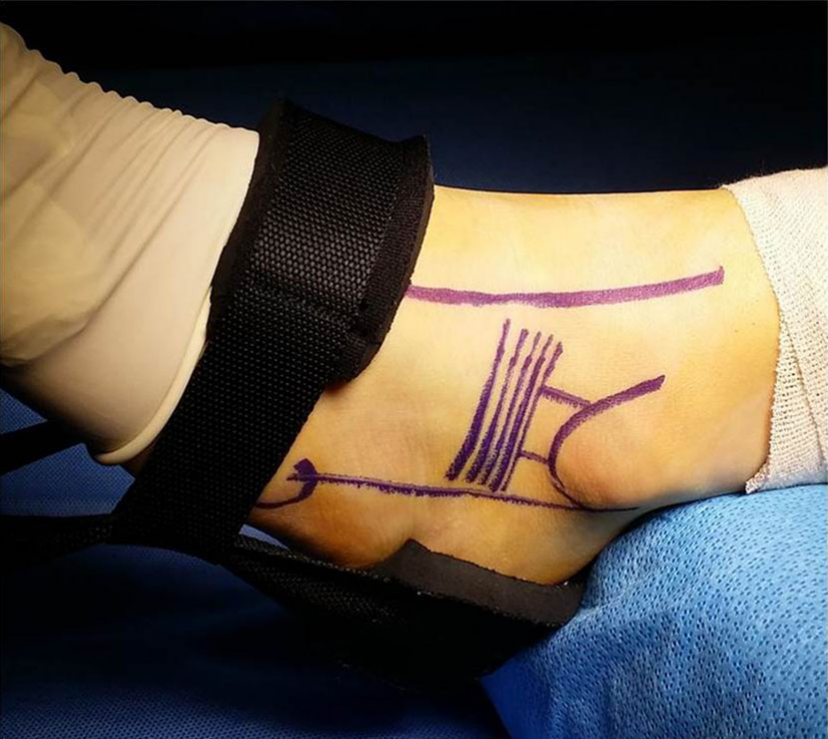
Preoperative anatomic landmarks (superficial peroneal nerve, superior border of the peroneal tendons, distal fibula anterior talofibular ligament, inferior retinaculum)

All patients were operated on by a single fully trained orthopedic surgeon (JSY). The patient was placed on the operating table in a supine position, and spinal anesthesia was administered. A well-padded thigh tourniquet was applied, and a thigh holder was positioned to elevate the foot a few inches off the operating table. It is imperative to outline the distal fibula, the course of the peroneal tendons, the superficial peroneal nerve, the anterior talofibular ligament and the inferior retinaculum with a surgical marker before initiating the procedure (Fig. 1).

Next, a noninvasive ankle joint distractor was applied and, under manual tension, the joint was slightly distracted. Ankle joint arthroscopy with standard anteromedial and anterolateral portals was performed. Any concomitant procedures were performed to address intra-articular pathologic features before proceeding with the lateral ankle stabilization. The anterolateral portal becomes the access point to the distal anterior fibula for anchor placement. Each step was visualized with a 30-degree arthroscope inserted through the anteromedial portal.

**Fig. 2:**
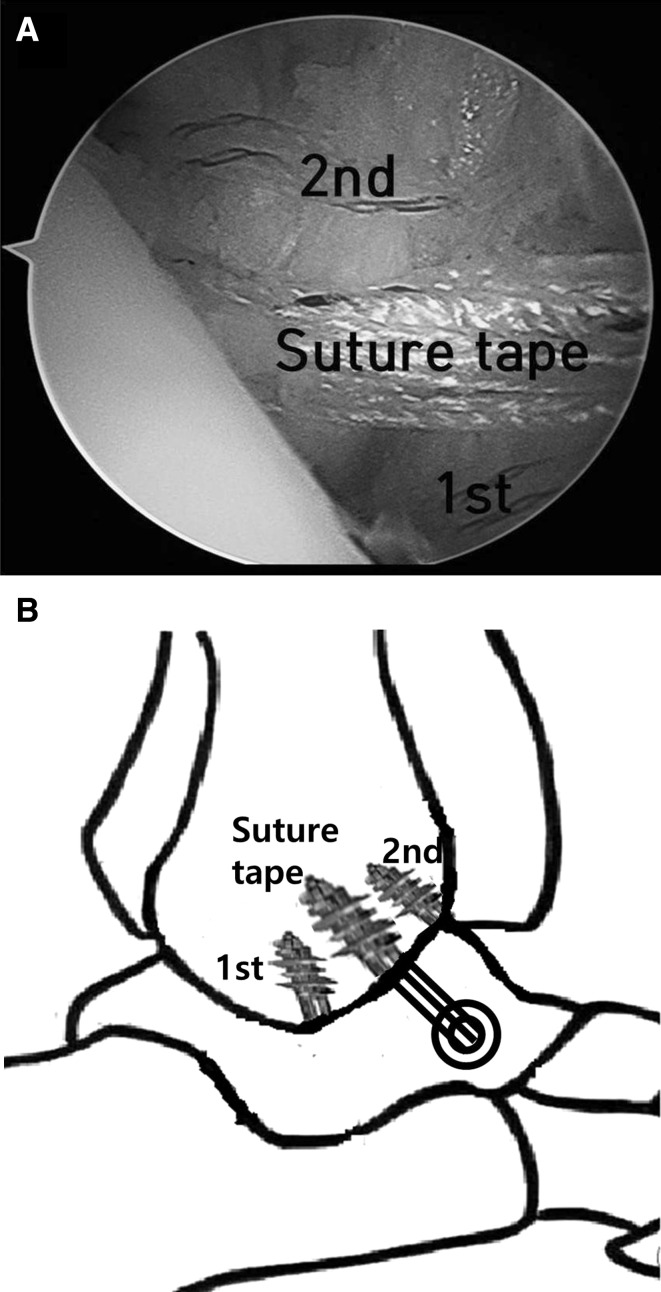
**a** Arthroscopic images demonstrating use of anterolateral portals for anchor placement. The first anchor was inserted at 1 cm superior to its position on the fibula. The second anchor was placed into the fibula more superiorly and level with the lateral shoulder of the talus. The fibular tunnel was created for suture tape insertion in the fibula between two all-suture anchors through the anterolateral portal. **b** Schematic drawing of an arthroscopic modified Brostrom procedure with an internal brace

Preparation for the first of two all-suture anchors was performed by inserting the drill guide through the anterolateral portal and held in position directly midline and approximately 1 cm superior to its position on the fibula in order to facilitate anchor placement. The first anchor was inserted through the drill guide and seated into position with a mallet. The handle and drill guide were removed, and the sutures exited through the anterolateral portal. A second anchor was then placed using the same technique. Ideally, this anchor should be placed into the fibula more superiorly and level with the lateral shoulder of the talus. Suture tape augmentation was then performed for internal bracing. A 3.4-mm tunnel was created in the fibula between two all-suture anchors through the anterolateral portal under arthroscopic view using a calibrated drill guide followed by a 4.75-mm tap (Arthrex Inc., Naples, FL, USA). A 4.75-mm suture anchor (BioComposite SwiveLock; Arthrex Inc.) was loaded with suture tape composed of braided ultra-high-molecular-weight polyethylene and polyester (FiberTape; Arthrex Inc.) and seated into the fibula (Fig. 2).

**Fig. 3:**
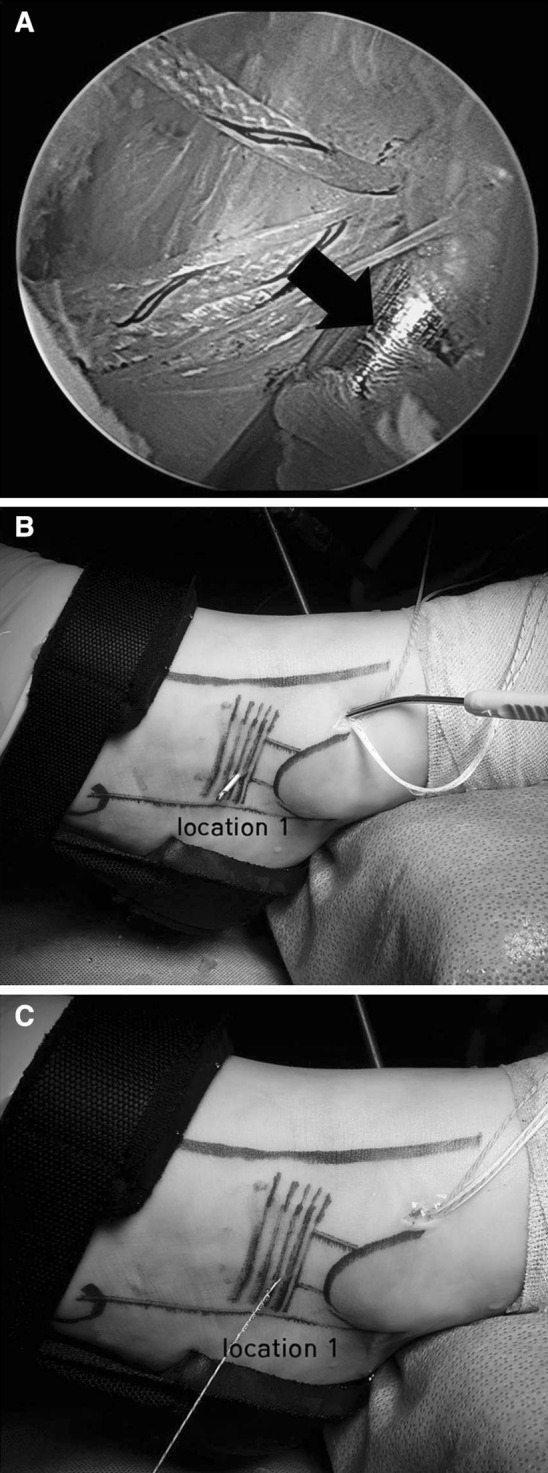
**a** Arthroscopic view of the banana lasso that passed through the anterolateral portal. Arrow indicates the banana lasso. **b**, **c** Photographic images show that the first pass was placed approximately 1 cm anterior and inferior to the distal anterior fibula

A banana lasso was then used to capture the residual ATFL, ankle capsule, and inferior extensor retinaculum. The first pass was placed approximately 1 cm anterior and inferior to the distal anterior fibula through the anterolateral portal. Caution was taken to avoid the sural nerve and peroneal tendons. The lasso was placed deep enough to capture the capsule, any residual ATFL, and the inferior extensor retinaculum under arthroscopic view (Fig. 3a). The nitinol wire was then advanced and used to capture one strand of the anchor suture, which was then pulled to exit the skin at location 1 (Fig. 3b). The second pass was placed approximately 1 cm distally and directed in the same manner though the anterolateral portal. The lasso was used to pull the second suture strand through the skin to location 2. The sutures exited the portal, and the banana lasso was used to individually capture each strand exiting the skin at 1 cm superior and anterior to the previous strand for location 3 and again for location 4. This creates a construct with four strands exiting the skin in 1-cm increments and placed to capture as much of the retinaculum and capsule as possible (Fig. 3c).

**Fig. 4:**
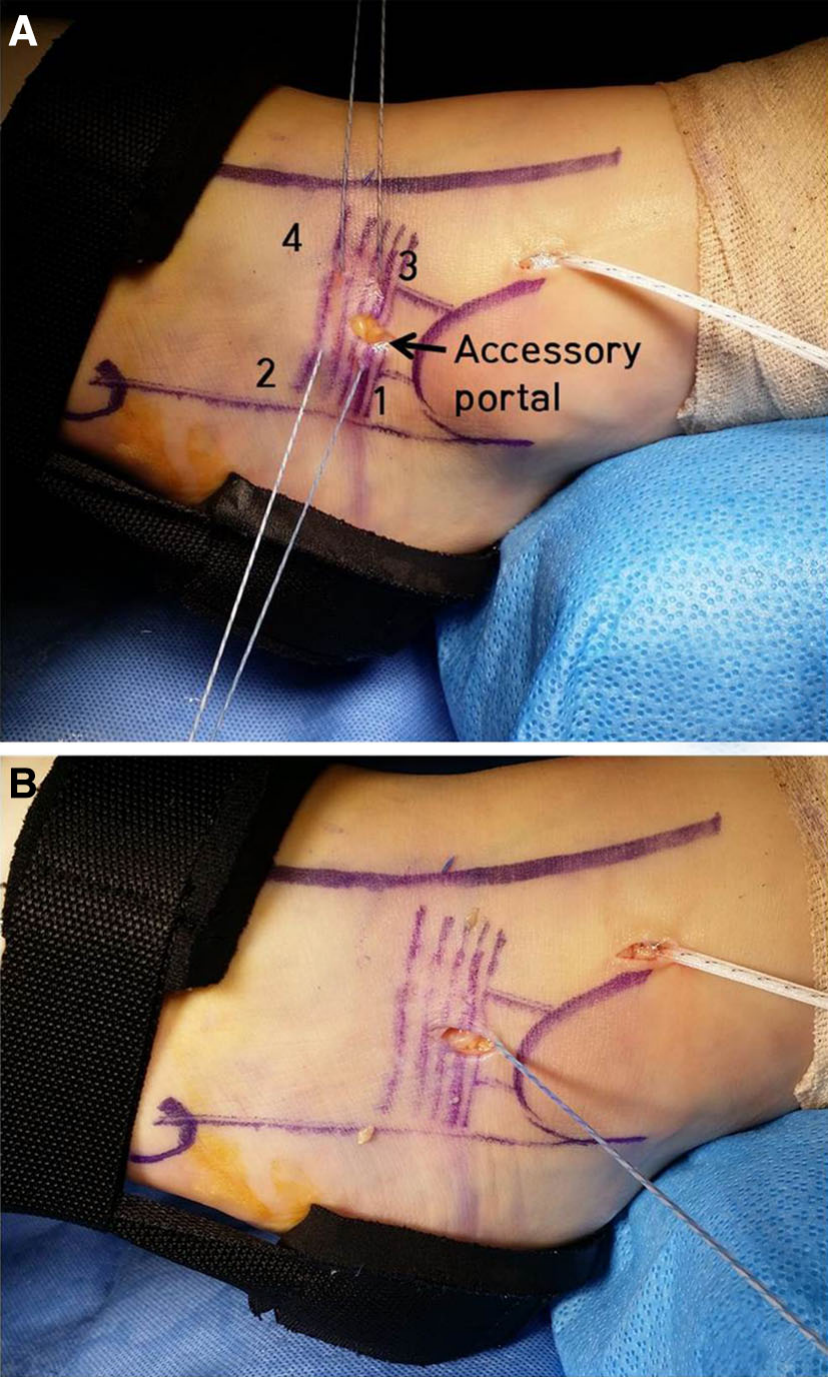
**a** A small accessory portal was made between the two sets of sutures. **b** A probe was introduced into the incision and used to subcutaneously gather the sutures, pulling them out through this accessory incision

A small accessory portal was then made between the two sets of sutures (between strand locations 1, 2 and 3, 4) (Fig. 4a); this was 1 cm in length, and only the skin was incised. A probe was introduced into the incision and used to subcutaneously gather the sutures, pulling them out through this accessory incision (Fig. 4b). Care was taken to keep each suture set together and avoid mixing between the two anchors. The foot was then released from distraction and held in an everted and slight neutral to dorsiflexed position. Before tying the sutures, we have found it imperative to clear any subcutaneous adipose tissue that might prevent the sutures from laying directly on the retinaculum. Surgical knots were placed and tensioned for each suture set, correlating to their respective anchor within the fibula. The suture ends were cut and the incisions closed in standard fashion.

**Fig. 5:**
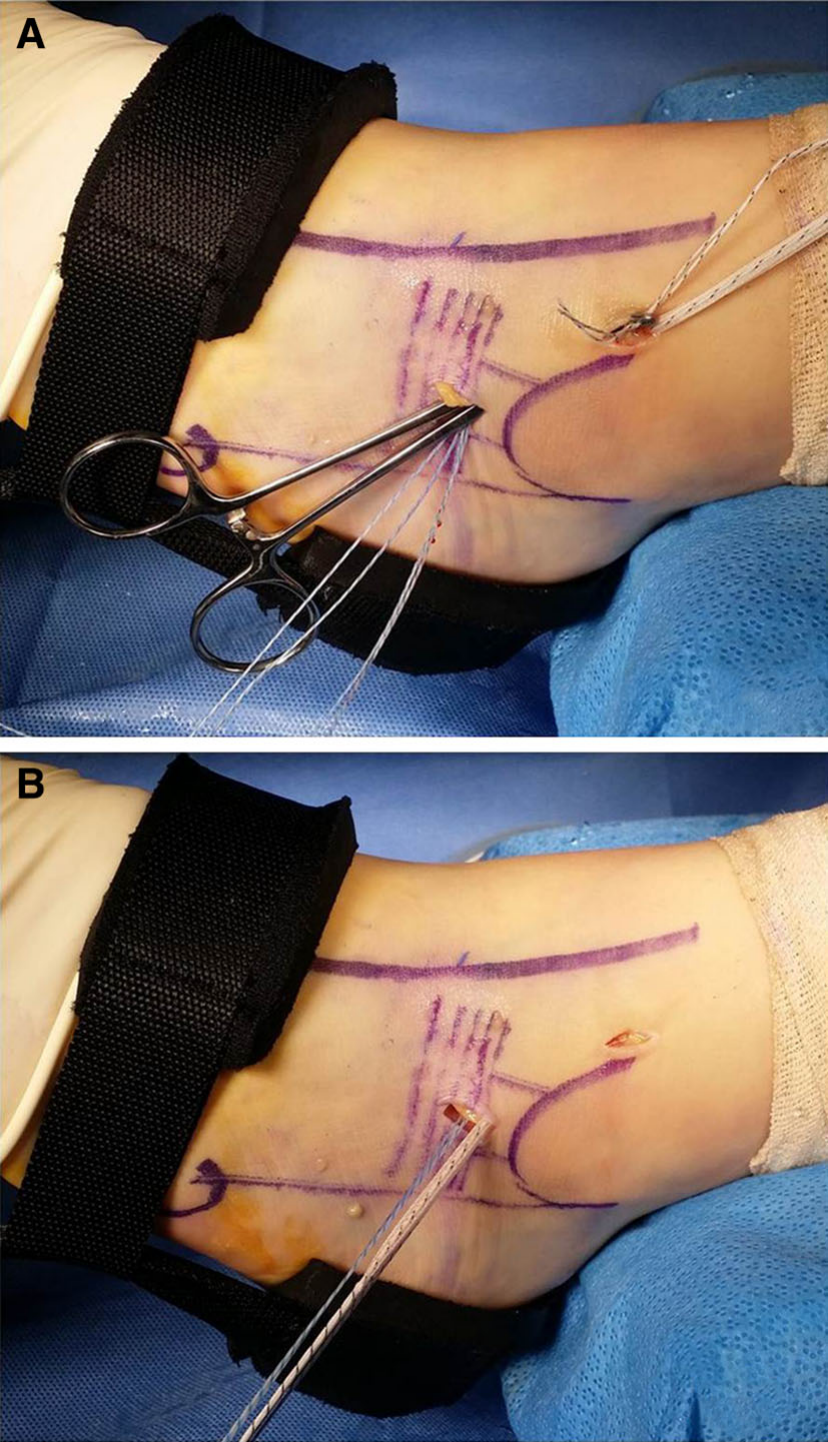
**a** The end of the suture tape was captured using a mosquito from the accessory portal to the anterolateral portal. **b** Photograph shows suture tape moved subcutaneously from the anterolateral portal to the accessory portal

**Fig. 6:**
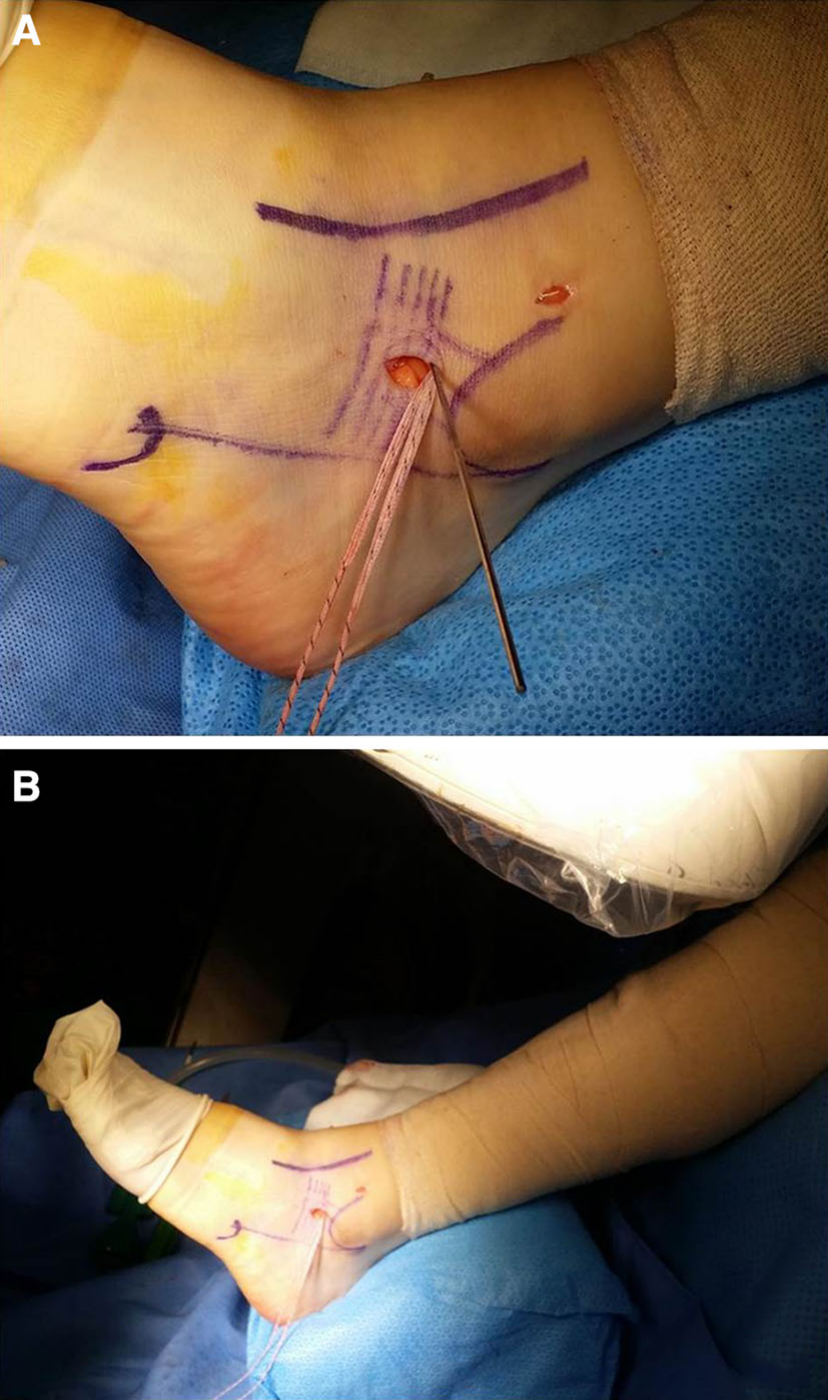
Another tunnel was created in the talus for insertion of the anterior talofibular ligament through the accessory portal. **a** Before creation of the tunnel, a Kirschner wire was inserted in the talus for insertion of the anterior talofibular ligament. **b** The position of the tunnel was confirmed under fluoroscopy

After tying the sutures of all-suture anchors, the suture tap was moved subcutaneously from the anterolateral portal to the accessory portal using the mosquito (Fig. 5). Another 3.4-mm tunnel was created at the talus of insertion of the native ATFL through the accessory portal under fluoroscopy, using a calibrated drill guide followed by a 4.75-mm tap (Arthrex Inc.) (Fig. 6). A second 4.75-mm anchor loaded with the opposite end of the suture tape was then seated into the talus under tension. The foot was then held in relaxed plantar flexion with a bump placed under the tibia to avoid overtightening.

The patients undergoing arthroscopic modified Brostrom operation without an internal brace were treated with the same technique as described above but without the use of an internal brace.

### Postoperative rehabilitation

#### Arthroscopic modified Brostrom operation with an internal brace group

After the operation, a compression bandage was applied without a splint and progressive weight-bearing was allowed. At 2 weeks, physical therapy including proprioceptive training, active ankle extension, and eversion exercises was started. Running and return to high-contact sports (soccer and basketball) were allowed at 4 weeks.

#### Arthroscopic modified Brostrom operation without an internal brace

After the operation, the ankle was immobilized in a short leg cast, and no weight-bearing was allowed for 2 weeks. Progressive weight-bearing was allowed after 2 weeks. At 4 weeks, the short leg cast was removed and a semi-rigid brace was applied. At 6 weeks, physical therapy, including proprioceptive training, active ankle extension, and eversion exercises was started. Running and return to high-contact sports (soccer and basketball) was allowed at 3 months.

### Clinical assessment

#### Arthroscopic modified Brostrom operation with an internal brace group

Clinical assessment was performed retrospectively. Patients were assessed preoperatively and at 1, 2, 6, 12 and 24 weeks after surgery. The American Orthopaedic Foot & Ankle Society (AOFAS) score was used to assess the functional status [15]. At preoperation and at 24 weeks after surgery, the anterior drawer test was examined clinically for instability evaluation. Instability was classified as normal (grade 0) in patients with <5 mm translation compared with the uninjured side, grade 1 in patients with 5–10 mm side-to-side difference, grade 2 in patients with 10–15 mm of side-to-side difference, and grade 3 in patients with >15 mm of difference.

#### Arthroscopic modified Brostrom operation without an internal brace

Patients were assessed preoperatively and at 6, 12, and 24 weeks after surgery. AOFAS score and clinical anterior drawer test were examined as above.

### Statistical analysis

SPSS (version 19.0, 2010; SPSS, Inc. Chicago, IL, USA) was used for statistical analysis. Paired data analysis correlated with the clinical evaluation was performed to compare improvement between the preoperative and postoperative score and to compare between the two groups. Comparison of the results using the AOFAS score was made by Wilcoxon signed rank test. Chi-squared test, Fisher’s exact test and Mann–Whitney test were used for comparing results between the two groups. Differences were considered statistically significant when *p* value was ≤0.05.

## Results

### Arthroscopic modified Brostrom operation with an internal brace group

The mean AOFAS score was 65.8 ± 21.8 (range 24–92) preoperatively, 70.6 ± 19.8 (44–87) at 1 week, 85.5 ± 20.7 (66–97) at 2 weeks, 95.9 ± 20.2 (87–100) at 6 weeks, 96.9 ± 19.4 (87–100) at 12 weeks, and 98.0 ± 16.8 (90–100) at 24 weeks. Improvement of mean AOFAS score from before surgery to 1 week after surgery was not statistically significant (*p* = 0.068). However, improvement of AOFAS score from before surgery to 2 weeks after surgery was statistically significant (*p* < 0.001). At 6-week follow-up, all patients returned to their daily activities without difficulties. At 12-week follow-up, 18 patients (81.8 %) returned to sports activity without limitations. At 24-week follow-up, the anterior drawer test showed grade 0 laxity in 19 patients (86.4 %) and grade 1 in three patients (13.6 %) (Table 1).

**Table 1:** Clinical results of the patients

	Mean AOFAS score						Mean grade of anterior drawer test	
	Preoperation	1 week	2 weeks	6 weeks	12 weeks	24 weeks	Preoperation	24 week
Internal brace group	65.8 ± 21.8	70.6 ± 19.8	85.5 ± 20.7	95.9 ± 20.2	96.9 ± 19.4	98.0 ± 16.8	2.7 ± 0.7	0.1 ± 0.4
Without internal brace group	66.7 ± 15.0			72.5 ± 13.0	92.0 ± 7.6	96.5 ± 5.4	2.6 ± 0.5	0.1 ± 0.4
*p* value	0.958			<0.001	<0.001	0.375	0.521	0.882

The other concomitant intra-articular findings were synovitis in 22 patients (100 %), anterior tibial spurring in one patient (4.5 %), and loose bodies in one patient (4.5 %). Two of the patients (9 %) presented signs of an inversion deficit of >10 degrees in the ankle compared to the contralateral side. No patient experienced wound dehiscence and/or infection, paresthesia, or numbness in their foot.

### Arthroscopic modified Brostrom operation without an internal brace

The mean AOFAS score was 66.7 ± 15.0 (range 44–92) preoperatively, 72.5 ± 13.0 (44–97) at 6 weeks, 92.0 ± 7.6 (52–100) at 12 weeks, and 96.5 ± 5.4 (68–100) at 24 weeks. Improvement of AOFAS score from before surgery to 6 weeks after surgery was statistically significant (*p* < 0.001). At 12-week follow-up, 17 patients (27.0 %) returned to sports activity without limitations. At 24-week follow-up, the anterior drawer test showed grade 0 laxity in 54 patients (85.7 %) and grade 1 in 9 patients (14.3 %) (Table 1).

The other concomitant intra-articular findings were synovitis in 58 patients (92.1 %), and loose bodies in two patients (3.2 %). Three of the patients (4.8 %) showed an inversion deficit of >10 degrees in the ankle compared to the contralateral side. Two of the patients (3.2 %) presented signs of neuritis of the intermediate dorsal cutaneous nerve; one of them showed full recovery after a steroid injection but the symptoms of the other patient persisted until the final follow-up. No patient experienced wound dehiscence.

### Comparison between the two groups

The AOFAS score at preoperation and at the final follow-up (24 weeks after surgery) showed no difference between the patients with an internal brace and those without an internal brace (*p* = 0.375). However, the AOFAS score at 6 weeks and at 12 weeks after surgery showed a significant difference between the two groups (*p* < 0.001) (Table 1). Furthermore, the rate of returning to sports at 12 weeks after surgery showed a significant difference between the two groups (*p* < 0.001). There was no difference between anterior drawer test and rate of complications (*p* = 0.882).

## Discussion

To date, the open modified Brostrom operation has been the gold standard procedure, with good-to-excellent results [16, 17]. Brostrom advocated a method of ankle ligament reconstruction in 1966 [2]; however, Gould later modified this technique by reinforcing the ligament with the inferior extensor retinaculum [17]. Theoretically, inferior extensor retinaculum reinforcement covers the calcaneofibular ligament vector. Furthermore, substantial initial stability was obtained using an anatomical reconstruction of the anterior talofibular ligament alone with inferior extensor retinaculum reinforcement [18].

Lee et al. [19] performed a review of simultaneous ankle joint pathologic entities for chronic lateral ankle instability. They reviewed 28 ankles that underwent ankle joint arthroscopy with concomitant open Brostrom−Gould stabilization and reported a frequency of 7–100 % for associated intra-articular pathologic features. Of the 28 ankles reviewed, 100 % were found to have some degree of synovitis, which was frequently identified in the anterolateral aspect of the joint. Other associated pathologic features were talar dome osteochondral defects in two ankles (7 %), talar dome fibrillation in seven (30 %), loose bodies in three (11 %), Bassett’s lesion in two (7 %), anterolateral impingement in four (14 %), and distal anterior tibial spurring in four (14 %). Ferkel and Chams [11] reported on 21 ankles that underwent ankle arthroscopic evaluation before a Brostrom−Gould procedure. They identified pathologic intra-articular findings in 95 % of their patients. Therefore, an arthroscopic inspection is almost mandatory because of the high incidence of concomitant intra-articular lesion [20]. A reliable arthroscopic method for treating ankle instability without the need for open surgery would be ideal [21].

Many studies have been reported on the strength and the clinical results of the arthroscopic modified Brostrom operation. Lee et al. [22] reported that there was no significant difference in torque to failure between the open and arthroscopic modified Brostrom operation through a biomechanical study of 11 human cadaveric specimens. In 2011, Nery et al. [23] reported the long-term results of an arthroscopic modified Brostrom operation in 38 patients with a mean follow-up of 9.8 years. The mean AOFAS score was 90 and only one patient required soft-tissue debridement for anterior impingement postoperatively. Corte-Real and Moreira [21] reported a similar technique but differed in that only one anchor was placed into the fibula, and only one distal location was used for the sutures to exit through an accessory portal. They followed up 31 patients for a mean 24.5 months and found an average postoperative AOFAS score of 94.4.

Moreover, Viens et al. [24] reported that the strength and stiffness of the Brostrom repair with suture tape augmentation were not significantly different from those of the intact ATFL in a cadaveric model. Prior research has reported ATFL with the standard Brostrom repair to be at least 50 % weaker than native ATFL at time zero [4]; the results of this study also show that suture tape augmentation techniques produce stronger and stiffer results than those of the standard Brostrom repair.

According to our results, the patients who underwent the Brostrom repair with an internal brace were allowed early rehabilitation without the need of early protection. Improvement of AOFAS score from before surgery to two weeks after surgery was statistically significant in the patients with an internal brace (*p* < 0.001), whereas improvement of AOFAS score from before surgery to six weeks after surgery was statistically significant in the patients without an internal brace (*p* = 0.001). Moreover, the AOFAS score at 6 and 12 weeks after surgery showed a significant difference between the two groups (*p* < 0.001).

In our study, two patients (9 %) with an internal brace presented signs of an inversion deficit of >10 degrees in the ankle compared to the contralateral side. Therefore, suture tape augmentation should be performed cautiously without overtightening. To avoid overtightening, the ankle should be positioned in the neutral position. Marking the distance between the original site of the fibula and the insertion site of the talus on the suture tape can also be useful.

There are several limitations to this study. The number of cases was small, and this was a retrospective study. Additional randomized comparative prospective studies are necessary. Additionally, as we did not perform stress radiographs, the functional outcomes were subjectively reported by the patients, and the objective findings were noted by us, physician bias might have influenced the outcomes.

Offering the soldiers or the athletic and recreational sports enthusiast a minimally invasive, virtually arthroscopic reconstruction with internal bracing would be advantageous. Because of the significantly smaller incisions, the arthroscopic technique provides a lower chance of wound dehiscence and complications compared with an open procedure. Our patients did not develop any wound complications, which enabled a quick return to activity and sports. We believe this technique could be a viable option in surgically treating chronic lateral ankle instability in those patients who need an early return to activity and sports.
